# Research Progress and Teaching Exploration of Physical Processing Technology for Reduced-Salt Gel Meat Products

**DOI:** 10.3390/foods13223606

**Published:** 2024-11-11

**Authors:** Zhuangli Kang, Qin Hou, Jingguo Xu

**Affiliations:** 1 School of Tourism and Cuisine, Industrial Engineering Center for Huaiyang Cuisin of Jiangsu Province, Yangzhou University, Yangzhou 225127, China; 008140@yzd.edu.cn (Q.H.); xu_jingguo@yzu.edu.cn (J.X.); 2 Key Laboratory of Chinese Cuisine Intangible Cultural Heritage Technology Inheritance, Ministry of Culture and Tourism, Yangzhou 225127, China

**Keywords:** physical processing, high-pressure processing, ultrasonic technology, reduced-salt gel meat, teaching reform, protein conformation

## Abstract

Salt assumes a significant role in the production of meat gels. Excessive intake of salt adversely affects human health, and consumers’ demand for reduced-salt meat products is escalating. This review primarily introduces the characteristics of the physical processing technology of reduced-salt gel meat products, such as the technology of ultrasonic, high-pressure processing, beating, plasma, and magnetic field, and its role in reduced-salt gel meat processing, and explores means to improve the teaching effect of the physical processing technology of reduced-salt gel meat products in the major of Food Science and Engineering. It was found that physical processing techniques, such as ultrasound, high-pressure processing, and beating, could enhance the solubility and processing performance of myofibrillar protein by improving the meat structure and protein conformation, increasing the interaction between proteins, water, and fat molecules, and enhancing the texture, water-holding capacity, and sensory quality of reduced-salt gel meat products. In the promotion and teaching of physical processing technology, it is necessary to strengthen interdisciplinary integration and scientific research activities according to the customs, laws and regulations of different countries and regions, combined with the development frontier of the technology, and develop reduced-salt gel meat products that meet local needs according to local conditions.

## 1. Introduction

Gel meat products, such as emulsified sausages and meatballs, are significant deep-processed meat products characterized by freshness and juiciness, diverse flavors, strong regionality, and high cost performance [1,2]. The degree of mechanization is elevated, and the output is substantial, accounting for approximately 50% of the total gel meat products [3]. Gel formation constitutes an essential link in the processing of gel meat products. Its formation is closely associated with the dissolution, aggregation, and cross-linking of myofibrillar protein. It is also a significant course in the undergraduate teaching of the food processing speciality [4,5]. Myofibrillar protein is the predominant protein component in meat, accounting for roughly 50% of the total protein. Under specific conditions, myofibrillar protein can form a gel network, conferring good texture and taste to meat products [6,7]. In actual production, approximately 2.0% salt is typically added, which facilitates the dissolution of myofibrillar protein in meat. For instance, the addition of salt causes meat fibers to hydrate and expand, and myosin dissociates from the myofibrillar protein bundle, thereby enhancing the water-holding capacity of the product [8,9]. Simultaneously, the addition of salt elevates the ionic strength of the meat batter, increases the molecular repulsion between meat fibers, and improves the solubility and processing properties of myofibrillar protein [10]. Additionally, alterations in ionic strength will influence the existing state of myosin and subsequently affect the aggregation of protein molecules. Therefore, as a salt-soluble protein, myofibrillar protein can be effectively dissolved and solubilized under high salt conditions (>0.3 mol/L) to form an excellent gel structure [8]. Salt is an indispensable condiment in daily life. Its principal component is sodium chloride, which plays a crucial role in maintaining the normal physiological functions of the human body [11]. For example: sodium ions in salt assist in maintaining fluid balance and ensuring that body tissues and organs receive adequate water; the body requires sodium for transmitting electrical signals to support nerve and meat functions; an appropriate amount of salt can promote the secretion of gastric acid, thereby facilitating digestion; proper salt intake can help maintain normal blood pressure [12,13,14]. The World Health Organization recommends that the daily salt intake of adults should not exceed 5 g. However, excessive salt intake has adverse effects on health. If an excessive amount of salt is consumed, it will lead to increased arterial pressure, which can result in hypertension. Simultaneously, with the rise in blood pressure, the pressure on the heart increases and cholesterol and blood lipids ascend, which is prone to increasing the risk of heart disease, coronary heart disease, heart failure, stroke, and other diseases [15,16,17]. Therefore, in food processing and daily diets, the amount of salt should be rationally controlled to maintain human health.

Previous studies have revealed that the intake of sodium chloride by residents from meat products accounted for about 20% of the total intake [3,18]. Reducing the quantity of salt added to meat products, particularly gel meat products, was beneficial for reducing consumers’ intake of sodium chloride. The classical gel meat theory holds that myosin must be extracted at a sufficiently high concentration of sodium chloride and fully dissolved to form a superior gel structure [19,20]. Directly lowering the concentration of sodium chloride reduces the extraction and dissolution of myosin and deteriorates the heat-induced gel structure. Hence, how to reduce sodium chloride while ensuring the quality of gel meat products poses a challenge.

The physical processing method is a technical approach to enhance the processing performance of gel meat through physical means (such as sound, light, electricity, magnetism, and force), including high-pressure processing, beating technology, plasma technology, microwave technology, magnetic field technology, etc., and it is also the knowledge that students majoring in food processing must acquire [21]. Previous studies have indicated that high-pressure processing, ultrasonic treatment, beating technology, plasma technology, and magnetic field technology can effectively enhance the processing performance of reduced-salt meat protein, and improve the gel quality and yield [22,23,24,25]. Moreover, physical processing does not require the addition of non-meat substances, which is conducive to achieving the “clean label” of gel meat products and is more acceptable to consumers [26]. In the contemporary higher education system, in the face of the diversification of students’ needs and the rapid advancement of Food Science and Technology, particularly in the cutting-edge domain of reduced-salt gel meat products processing technology, the innovation of the teaching mode is exceptionally crucial. How to adjust teaching strategies to satisfy students’ dual pursuit of professional depth and breadth as well as industry foresight constitutes a challenging and hot topic in research. To broaden the application of physical processing methods in reduced-salt gel meat products, the aim of this study is mainly to review the mechanism of physical processing methods in improving the quality of reduced-salt gel meat products and explore its approaches in the process of undergraduate teaching.

## 2. The Physical Processing Technology of Reduced-Salt Gel Meat Products

### 2.1. Ultrasonic Technology

Ultrasound is a green physical processing technology for food, which possesses the advantages of being non-toxic, having simple operation and high efficiency. It plays a crucial role in food modification, meat tenderization, microbial inactivation, rapid freezing, thawing and drying [27,28]. The application of ultrasound in food can be classified into low-frequency high-intensity ultrasound (20–100 kHz, 10–1000 W/cm^2^) and high-frequency low-intensity ultrasound (1–100 MHz, <1 W/cm^2^) based on its frequency and intensity. In recent years, low-frequency high-intensity ultrasound has become a hotspot in the field of food physical processing technology by altering the structural properties of protein through the cavitation effect and subsequently enhancing the functional characteristics of various food proteins and product quality properties. The effect of food protein modification is mainly attributed to the instantaneous spatio-temporal variation generated by ultrasound. Because the cavitation bubble expands rapidly under ultrasound and collapses when it reaches the resonance critical point, it can locally produce an instantaneous high temperature of up to 5000 K and a high pressure of 30 MPa [29,30]. The physical effects caused by ultrasonic cavitation mainly comprise strong shear force, high pressure, shock wave, micro jet, turbulence and acoustic flow. These physical effects are mingled together in the ultrasonic treatment process, which can enhance the mass transfer effect, cause the folding and unfolding of protein molecules, and have a significant influence on protein–water interaction and protein–protein interaction [31,32]. Previous research has reported that the cavitation effect of ultrasound mainly affects the secondary and tertiary structure of protein (Table 1). This is due to the mechanical vibration and turbulence produced by the collapse of bubbles, which increases the chance of collision between protein molecules. The increase in α-helix content indicates that the protein structure becomes more compact under ultrasound. Under the action of ultrasonic cavitation, these structures are transformed into each other to a certain extent, leading to the improvement of the processing performance of wooden breast meat and other products [33,34,35]. Furthermore, ultrasound technology in combination with basic amino acid, sodium bicarbonate, TGase, and other materials to improve the gel properties and cooking yield of reduced-salt meat batters were reported [27,36,37]. Kang et al. [27], regarding the utilization of ultrasound and sodium bicarbonate combination on the reduced-salt pork batters, found that the method could enable reduced-salt samples to have better gel characteristics and higher cooking yield. Huang et al. [38] found that ultrasound assists *L*-lysine/*L*-arginine to improve the quality characteristics and decrease the cooking loss and total expressible fluids of emulsion sausages. Therefore, it can be seen from the above that ultrasonic technology alone or combined with other non-meat additives can effectively improve the processing performance and product quality of reduced-salt meat batter products.

### 2.2. Plasma Technology

Plasma is the fourth form existing alongside the solid, liquid, and gas states. It is a neutral gas composed of a large number of free radicals, positive and negative ions, electrons, and neutral particles generated during the process of gas discharge [39]. Plasma is categorized into high-temperature plasma and low-temperature plasma. High-temperature plasma demands an extremely high temperature, which is difficult to achieve in general laboratories and is thus rarely employed; on the other hand, low-temperature plasma can be further divided into thermal plasma and cold plasma. The temperature of thermal plasma is high, while that of cold plasma is close to room temperature. Generally, cold plasma is also referred to as low-temperature plasma and is utilized in the majority of laboratories. In recent years, low-temperature plasma sterilization technology has been gradually applied in the food industry [39,40]. The advantages of low-temperature plasma sterilization primarily encompass a short action time, low energy consumption, an excellent sterilization effect, and no obvious impact on food quality (Table 2). Additionally, the gas surrounding the food treated by low-temperature plasma also possesses bactericidal effects, which are more conducive to the storage of food. Ekezie et al. [41] discovered that the pH and protein solubility of myofibrillar proteins from king prawns decreased while the temperature increased with plasma treatment time. Jung et al. [42] reported that atmospheric pressure plasma gradually raised the temperature of pork meat batter (1% sodium chloride) over 60 min from 0.2 to 20 °C, and the a*- and b*-values gradually increased and decreased, respectively, with the increase in treatment times. Qian et al. [43] found that due to the active ingredients in plasma active water (treating distilled water with plasma), the environmental polarity of amino acids can be changed, protein intramolecular/intermolecular interactions can be enhanced, and the generation of disulfide bonds can be induced, subsequently breaking the inherent charge balance of myofibrillar protein molecules, resulting in the enhancement of protein cross-linking and further promoting the formation of a dense and solid chicken myofibrillar protein gel network structure. Rao et al. [44] also discovered that the plasma-activated water contains multiple active species that modify the structure of myofibrillar protein to enhance their gel properties, which can be utilized to produce the reduced-salt gel meat product. Jiang et al. [45] reported that sodium bicarbonate can effectively enhance the processing performance of duck myofibrillar proteins during cold plasma treatment by increasing the active sulfhydryl content, enhancing the surface hydrophobicity, intensifying the intrinsic fluorescence intensity, and reducing the average particle size. In particular, it should be noted that the parameters must be reasonably controlled during the application of plasma technology to prevent excessive aggregation and denaturation of meat proteins, which could lead to the deterioration of protein gel properties.

### 2.3. Magnetic Field Technology

A magnetic field is generated by the movement of charges. In physics, magnetic field strength is employed to describe the magnitude of the magnetic field generated by current in a specific space. Based on the size and direction of the current, the magnetic field can be classified into static magnetic field and alternating magnetic field [46]. The magnetic field is a novel physical processing approach. As it is harmless to the human body and has a low processing cost, it has been extensively studied in life science and food in recent years. In the domain of food processing, previous studies predominantly applied the magnetic field to the preservation and storage of food. This is attributed to the characteristics that the magnetic field can alter the permeability of biofilms, influence the biochemical reactions of organisms, and inhibit the activity of biological enzymes (Table 3). Qian et al. [47] demonstrated that the magnetic field can cause the loss of activity of thermophilic bacteria, molds, and pectinase in orange juice. Lin et al. [48] utilized a static magnetic field to assist in the freezing treatment of beef, which maintained the integrity of the meat tissue to the greatest extent and played a significant role in enhancing the shelf life of beef and reducing dripping loss and cooking loss. In recent years, researchers have begun to employ magnetic field treatment to modify the protein structure of food, and to produce products with structural and functional characteristics distinct from natural proteins. Guo et al. [49] discovered that the low-frequency alternating magnetic field of 0.5 MT enhanced the water retention and rheological properties of pork myofibrillar protein gel. Zhao et al. [50] also found that the low-frequency alternating magnetic field significantly improved the microstructure and acceptability of low-salt ground pork gel. Yang et al. [51,52] confirmed that the DC magnetic field played a vital role in improving the quality of pork myofibrillar protein. Zhao et al. [53] reported that the low-frequency alternating magnetic field combined with calcium chloride could increase solubility, surface hydrophobicity, active sulfhydryl content, and water-holding capacity of low-salt myofibrillar protein, leading to the formation of a homogeneous and compact gel structure. The above shows that magnetic field has great potential in protein modification, but most of the existing literature focuses on animal protein, and there is little research on blood protein. In addition, many studies have confirmed that magnetic fields can indeed affect the structural and functional properties of proteins; however, the study of its mechanism still needs to be strengthened.

### 2.4. High-Pressure Processing

During high-pressure processing, the pressure levels typically exceed 100 MPa, with the commonly employed range being 100–1000 MPa, and it can operate within the temperature range of −20 to 90 °C. Once the food is sealed in an elastic container or placed in a pressure system, non-covalent bonds are destroyed or formed at a specific temperature for an appropriate processing time and pressure level [54,55]. This leads to the inactivation, denaturation, and gelatinization of enzymes, proteins, starch, and other biological macromolecular substances in food, respectively, and the killing of microorganisms in food organisms, thereby achieving the goals of food sterilization, preservation, and processing. It is essentially a physical process that utilizes a liquid medium. It possesses the advantages of uniform pressure transmission, instantaneity, efficiency, minimal pollution, and negligible effects on low molecular compounds [56,57]. Therefore, this technology shapes the appearance and develops new types of meat with diverse textures in meat processing and storage.

At present, the application of high-pressure technology in meat processing mainly encompasses improving meat quality and sterilizing, such as enhancing meat tenderness and gel properties. The processing effects on meat-based products highly depend on the primary impacts of pressure, time, and temperature on the relevant thermodynamic and transport properties of meat systems [55]. For reducing the salt content of gel meat products (Table 4), high-pressure processing has attracted attention in gel meat products as it meets consumers’ demands for reduced salt content [58]. Due to the significant increase in β-sheet, β-turn, and random coil content along with the decrease in α-helix content, the shift of the –OH stretching vibration to higher wavenumbers, and the increase in the number of hydrogen bonds in each water molecule from 0.1 to 300 MPa, the water-holding capacity, surface hydrophobicity, and reactive sulfhydryl group content of reduced-salt chicken batters have significantly increased [59]. Similarly, Yang et al. [60,61] found that high-pressure processing treatment reduced the drip loss of reduced-fat reduced-salt pork batter by altering α-helix, random coils and CH_3_, CH, O-H, and S-S stretchings. The results improved the tenderness and modified the water distribution characteristics of reduced-fat reduced-salt pork meat batters, such as the transition of free water to immobilized water and bound water. Sikes et al. [62] discovered that the use of pressure processing at 400 MPa for 2 min on beef sausages with low-salt content led to reduced cooking losses and improved texture properties. Zheng et al. [63] reported that the quality of low-salt chicken batter was enhanced by heating under 200 MPa, and pressure was a determining factor for pressurized samples. Moreover, high pressure, rather than salt, was the main factor influencing the quality of chicken meat batter when heated under high pressure. Previous studies revealed that high-pressure processing improves the sensory properties of sausage [64,65] Therefore, high-pressure processing technology can enhance the quality and sensory aspects of reduced-salt gel meat products.

### 2.5. Beating and Chopping Technology

**Figure 1:**
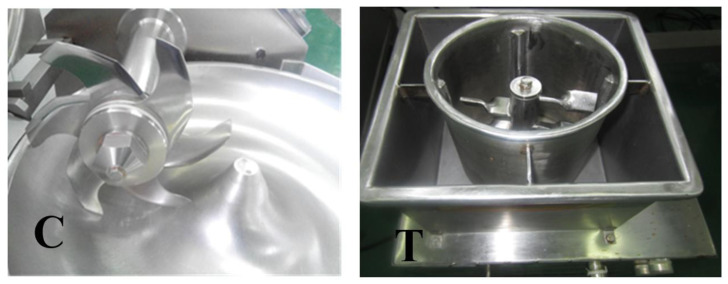
C: Chopping machine and knives; T: Beating machine showing blunt paddles. Reprinted with permission from Ref. [66].

**Figure 2:**
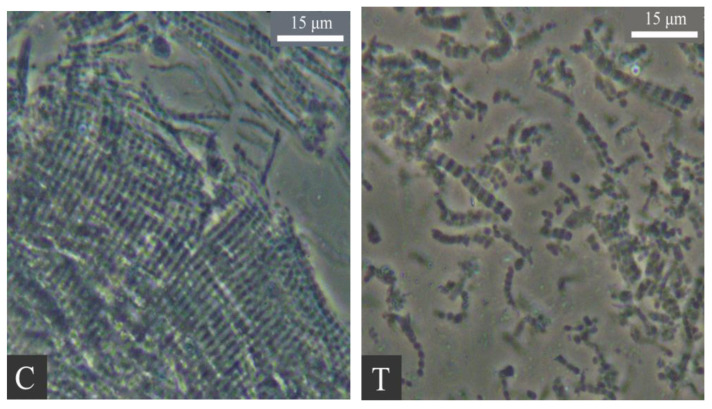
Phase-contrast micrographs of the myofibrils from the raw pork meat batters produced by either the chopping or beating process. Note: C, treatment with chopping process; T, treatment with beating process Reprinted with permission from Ref. [67].

It is a crucial link in the processing of gel meat products to crush the meat and obtain suitable ground meat particles. This procedure disrupts meat tissues such as myofibrils, meat intima, and meat fascial membranes, facilitating the hydrolysis and swelling of myofibrillar protein upon salt addition, accelerating the dissolution of myofibrillar protein and enhancing the quality of ground meat [66,67]. Chopping machines are widely utilized and stable crushing, mixing and emulsifying equipment in the actual production of gel meat products (Figure 1C). It mainly relies on the shearing force and tearing force generated by the rotation of the knives and the high-speed operation of the knives to cut the meat pieces and mix them evenly [23,67]. Beating machines are widely employed in China (Figure 1T), mainly for the production of gel meat products such as *Kongwan* and *Chaoshan* beef balls. The paddle is the principal component of a beating machine, which is conspicuously distinct from the sharp knife. The thickness of the paddle is generally greater than 0.5 cm, which determines the dissimilar principle of crushing meat between the chopping and the beating machine. Currently, the largest beating machine utilized by the company has a capacity of 800 L. During the beating process, the rotational speed of the paddle is generally greater than 100 rpm. A too-low rotational speed is detrimental to the crushing of meat and the dissolution and swelling of myofibrillar protein after salt addition. An overly fast rotational speed leads to a rapid increase in the temperature of ground meat, a reduction in processing time, and a decrease in the dissolution of myofibrillar protein, which is disadvantageous to the formation of the gel structure. Kang et al. [67] observed with a bright field phase contrast microscope and discovered that both chopping and beating can crush meat fibers, but the myofibril fragments in the chopping group are longer, and the myofibril fragments in the beating group are shorter, with the majority being less than 10 sarcomeres (Figure 2). The main peak and acromion were located at 20 μm and 138 μm in the chopping group, while those in the beating group were located at 15 μm and 91 μm, respectively, and the values of D_3,2_, D_4,3_, d_0.1_, d_0.5_ and d_0.9_ were significantly reduced. Scanning electron microscope results indicated that the meat bundles in the chopping group were neatly gathered, and the myofibrils and myofascial membranes remained intact. Numerous fine filaments were produced in the beating group, and many myofibrils and filaments were crosslinked together. Additionally, compared with the chopping group, the beating group has a lower α-helix content, a higher β-fold, β-turn and random coil content, a higher potential energy conformation, and more hydrophobic micro-groups are exposed to the polar aqueous solution, which causes the C-H stretching vibration spectrum band to shift towards the high wavelength direction. Owing to the more substantial disruption of myofibrils by the beating process, the solubility of myofibrillar protein is increased, which is conducive to the formation of a favorable gel structure. Compared with chopped ground meat with the same salt addition, the beating process can improve the L*-value, emulsification stability, texture, salt soluble protein solubility and end point G’, and reduce the Tanδ value of ground meat with 1% salt addition. The L*-value, hardness, elasticity and salt-soluble protein solubility of ground meat with 1% salt addition are higher than those with 2% salt addition, which are the same as those with 2% salt addition [23]. By altering the end point G’ and Tanδ values, it can be concluded that the beating treatment can mitigate the damage of myosin tail denaturation to the formed gel network structure during heat treatment [66].

## 3. The Promotion and Teaching of Reduced-Salt Gel Meat Products Physical Processing Technology

Although numerous papers have been published on the physical processing of reduced-salt gel meat products, there are few industrial applications. The main reasons are as follows: 1. Food practitioners have no understanding of the relevant technology and have no idea where to start when they want to apply it; 2. The new technologies have strict requirements on processing conditions, such as ultrasound processing, high-pressure processing, plasma, and magnetic field technology. Minor changes will have a significant impact on product quality; 3. The equipment is updated rapidly and the operation requirements are strict; 4. Customs, laws, and regulations in different regions vary, and standards are not uniform. For these reasons, in order to expand the application of this technology, it is necessary to provide technical training to potential users and lay a solid foundation for the development of new reduced-salt gel meat processing technologies adapted to local conditions. Therefore, the improvement and refinement of teaching methods in the promotion process are indispensable, which is conducive to the industrialization of the physical processing of reduced-salt gel meat products.

The improvement of promotion and teaching methods of reduced-salt gel meat products’ physical processing technology are the most important things to promote its development. In particular, with the swift advancement of science and technology and the intensification of global market competition, the promotion and teaching of reduced-salt gel meat products’ processing technology are encountering unprecedented challenges. To meet consumers’ demands for reduced-salt gel meat products, it is essential to enhance development and processing technology and cultivate technical personnel with a solid theoretical foundation and outstanding innovation and practical capabilities. Given the diverse nature of needs, the traditional solitary knowledge system is unable to fulfill the all-round development requirements of contemporary students. In the promotion and teaching of reduced-salt gel meat products processing technology, apart from the solid fundamental theoretical knowledge, it is necessary to integrate diversified contents such as industry case analyses, market trend research, and more [68]. For instance, students are organized to participate in the research and development projects of simulated cooperative enterprises, grasp professional skills during the process of resolving practical issues, and profoundly comprehend the alterations in market dynamics and consumer demands [69,70]. Simultaneously, interdisciplinary courses, such as food processing machinery, food nutrition, and marketing, are provided to broaden students’ knowledge, and enhance their comprehensive quality [71]. Given the rapid changes in reduced-salt gel meat products’ physical processing technology, a high degree of sensitivity and flexibility must be maintained throughout the teaching process. Teachers should constantly pay attention to the latest physical processing technology and scientific research, updates of technical standards and policies and regulations, and ensure the timeliness and cutting-edge nature of the teaching content [72]. At the same time, digital teaching tools, like online course platforms and virtual reality laboratories, should be employed to offer convenient learning resources and interactive communication platforms [73]. By inviting meat experts to hold lectures, workshops, and other forms, students can directly comprehend the industry status and trends of reduced-salt gel meat products. Additionally, an international perspective should be established to develop the physical processing technology for reduced-salt gel meat products suitable for the local market in accordance with the dietary habits and regulatory requirements of different countries and regions [74]. Moreover, in the promotion and teaching of reduced-salt gel meat products physical processing technology, the proportion of experiment courses is increased and various teaching activities, including case analysis and group discussion, are designed so that students can deepen their understanding of theoretical knowledge [73,75]. Students are encouraged to participate in scientific research projects, which can enhance their scientific research ability. Through internship training, enterprise cooperation, and other means, the effective connection of education, talent, industry, and innovation is achieved. The application of these teaching methods improves the teaching effect and lays a solid foundation for cultivating high-quality food processing technical talents to meet the demands of the industry.

## 4. Conclusions

In this paper, the impacts of physical processing technologies on the texture, color, and water-holding capacity of reduced-salt gel meat products were reviewed, and the teaching approaches of these technologies in Food Science and Engineering were explored. It was discovered that rational processing parameters and techniques could enhance the solubility and processing properties of meat protein under reduced-salt conditions, especially the alterations of the secondary and tertiary structures of the protein. Nevertheless, unreasonable physical processing can also result in the reduced processing performance of meat protein, such as decreased solubility, increased aggregates, and excessive cross-linking between molecules. In the promotion and teaching process, it is essential to adhere to the combination of theory and practice, pay attention to the development of cutting-edge technologies, strengthen school–enterprise cooperation, and enhance the teaching quality of reduced-salt gel meat product physical processing technology.

## Figures and Tables

**Table 1:** Effect of the ultrasound type on product properties of reduced-salt gel meat products.

Product	Ultrasound Type	Effect of Properties	References
Reduced-salt pork batters	Ultrasound-assisted sodium bicarbonate	The pH, protein solubility, cooking yield, and b* values of reduced-salt pork batters significantly increased with the increase in ultrasound time, and the use of ultrasound-assisted sodium bicarbonate treatment caused the batters to form a typical spongy structure with more evenly cavities.	[27]
Wooden breast meat batter	High-intensity ultrasound	High-intensity ultrasound effectively enhanced the water-holding capacity of wooden breast batters at 1% and 2% NaCl, and decreased the particle size of wooden breast batters at 1% NaCl, whereas opposite trends were observed at 2% NaCl. In addition, ultrasound treatment transformed into an α-helix structure to random coil at 1% NaCl, whereas it was transformed into a β-sheet structure at 2% NaCl.	[33]
Reduced-salt chicken breast meat batter	Ultrasound treatments with different salt contents	Ultrasound treatments improved the texture and water-holding capacity, and had higher G’ values. However, longer ultrasound treatment (40 min) resulted in a decrease in hardness, G’ value and water-holding capacity. Ultrasound treatment for 20 min lowered the values of spin–spin relaxation time and increased the proportion of myofribillar water.	[34]
Reduced-salt silver carp meat batter	Low-frequency ultrasound	Ultrasonic exposure decreased dense aggregates and increased the number and distribution of small cavity samples. Reduced-salt samples (1% salt) subjected to 30 min sonication had better color than 0 min sonication, better texture and cooking loss comparable to that of regular-salt level samples subjected to 30 min sonication, and similar to microstructures from normal salt samples without ultrasound exposure.	[35]
Reduced-salt pork myofibrillar protein	Ultrasonic-assisted sodium bicarbonate	Ultrasonic-assisted sodium bicarbonate significantly increased cooking yield, and texture properties of myofibrillar protein while reducing L*, a*, b*, and white values, centrifugal loss. Storage modulus significantly increased for myofibrillar protein treated with ultrasonic-assisted sodium bicarbonate treatment.	[36]
Reduced-salt chicken meat batter	Sonicated-assisted TGase	The pH, cooking yield, texture properties, β-sheet and β-turn contents of reduced-salt chicken meat batter considerably increased with increasing sonication duration, especially the cooking yield and hardness were increased from 80.7% and 58.5 N to 90.8% and 76.3 N.	[37]
Emulsion sausage	Ultrasonic and basic amino acid	Ultrasonic and *L*-lysine combined treatment resulted in a decrease in cooking loss, total expressible fluids, and expressible fat in sausages; ultrasonic and *L*-arginine combined treatment led to a decrease in cooking loss, total expressible fluids, and expressible fat. Ultrasonic treatment led to an increase in the storage modulus and apparent viscosity of meat batter.	[38]

**Table 2:** Effect of the plasma type on product properties of reduced-salt gel meat products.

Product	Plasma Type	Effect of Properties	References
Myofibrillar proteins from king prawn	Atmospheric pressure plasma jet (0, 2, 4, 6, 8 and 10 min)	The pH and protein solubility were decreased after 10 min treatment, and an increase in the mean particle diameter of myofibrillar proteins from 654 to 2297 nm. Thus, the high energetic and oxidizing effects have considerable implications on the physical and structural properties of myofibrillar proteins.	[41]
Pork meat batter	Atmospheric pressure plasma	Meat batter was treated with atmospheric pressure plasma during mixing. The temperature rose from 0.2 to 20 °C over 60 min plasma treatment, and the a*- and b*-values of cooked batter gradually increased and decreased, respectively, with incing the time.	[42]
Chicken myofibrillar protein gel	Plasma activated water	Plasma-activated water treatment accelerated the aggregation of chicken myofibrillar proteins and increased the strength and water-holding capacity of their gels, and the gels were endowed with antibacterial activity against *Salmonella Enteritidis* and *Staphylococcus aureus*.	[43]
Duck myofibrillar protein	Plasma activated water	Plasma-activated water alters the structure of myofibrillar proteins to enhance their gel properties. Plasma-activated water treatment caused the oxidation of 8241 cysteine sites on 2815 proteins, and structural proteins were susceptible to oxidation, and these proteins with differential oxidation sites were mainly derived from the cytoplasm and membrane.	[44]
Duck myofibrillar protein	Cold plasma with sodium bicarbonate	Cold plasma with sodium bicarbonate effectively mitigated the protein aggregation and induced secondary and tertiary structural alterations, enhancing the solubility of duck myofibrillar protein, and consequently improving their emulsifying and rheological properties.	[45]

**Table 3:** Effect of the magnetic field on product properties of reduced-salt gel meat products.

Product	Magnetic Field	Effect of Properties	References
Pork myofibrillar proteins	Low-frequency magnetic field (LF-MF; 0, 0.25, 0.5, and 1.4 mT)	The water-holding capacity and rheological properties of myofibrillar protein gels treated at 0.5 mT were better than those from other treatments. The ratios of immobilized water and free water significantly decreased and increased, respectively, with intensity increased, and the intensities of characteristic peaks in the tryptophan and aliphatic residues band were highest at 0.5 mT.	[49]
Low-salt pork batters	Low-frequency alternating magnetic field	Application of low frequency alternating magnetic field exhibited a significant improvement in the water-holding capacity, gel strength, color, textural properties and acceptability of low-salt pork batter gels. When the intensity reached 0.6 mT, the low-salt pork batters had the highest L*, gel strength, texture properties and acceptability.	[50]
Pork myofibrillar proteins	Direct current magnetic field	Water-holding capacity increased from 83.40% to 87.20% when direct current magnetic field treatment time changed from 1 h to 8 h. The 3 h treatment time promoted myofibrillar proteins unfolding, rearrangement and aggregation, thus a firmer and more ordered myofibrillar proteins gel network for trapping more water. But excessive treatment (8 h) weakened the direct current magnetic field effect on protein aggregation, gel network and texture.	[51]
Pork myofibrillar protein gels	Direct current magnetic field (3.5, 3.8, 9.5 and 10.4 mT)	Direct current magnetic field treatment significantly improved water-holding capacity compared with control, reaching the maximum value at 3.8 mT, and the increased intensity of tyrosine, aliphatic and tryptophan residues, and reduced reactive sulfhydryl. In addition, direct current magnetic field treatment helped to generate a relatively loose and uniform microstructure to trap more water.	[52]
Low-salt myofibrillar protein	Low frequency alternating magnetic field (5 mT, 3 h) and CaCl_2_ (0–100 mM)	Low frequency alternating magnetic field combined with 80 mM CaCl_2_ treatment increased solubility, surface hydrophobicity, active sulfhydryl content, and water-holding capacity, along with decreasing turbidity, particle size and intrinsic fluorescence strength. Thus, low-frequency alternating magnetic field combined with CaCl_2_ treatment can as a potential approach for modifying the gel characteristics of low-salt myofibrillar protein.	[53]

**Table 4:** Effect of the high-pressure processing on product properties of reduced-salt gel meat products.

Product	Ultrasound Type	Effect of Properties	References
Reduced-salt eucheuma spinosum chicken breast batters	High-pressure processing at 0.1 to 500 MPa for 10 min	Water-holding capacity, surface hydrophobicity, and reactive sulfhydryl group content significantly increased with the pressure increased from 0.1 to 300 MPa, and the β-sheet, β-turn, and random coil significantly increased with the α-helix decreased.	[59]
Reduced-fat reduced-salt pork batter	High-pressure processing at 100 to 400 MPa for 2 min	Total expressible fluid of reduced-fat reduced-salt batter reached its minimum value at 200 MPa, and pressure up to 200 MPa remarkably increased protein unfolding but 400 MPa increased aggregation.	[60]
Reduced fat and salt meat batters	High-pressure processing	The highest stability and activity of meat emulsion were obtained as 68.12% and 76.89% at 200 MPa, respectively, and the minimized diameter of emulsion particles was D_3,2_ 2.29 μm, and the diameter of fat droplets and proportion of ionic bonds first decreased then rose along with increasing pressure, and the lowest values were exhibited at 200 MPa.	[61]
Low-salt beef sausages	High-pressure processing at 400 MPa for 2 min	Unpressurised sausages containing 2% NaCl had a similar cook loss to pressure-treated sausages containing 1% NaCl. The hardness and gumminess of pressure-treated samples were higher compared to untreated samples at all salt concentrations.	[62]
Low-salt chicken batter	Heating under pressure at 0–400 MPa, at 75 °C, for 30 min	Regardless of salt content, the application of heating under pressure treatment at 200 MPa improved the gel qualities, resulting in a glossy, rigid gel with low water loss and high acceptability, while treatment at 400 MPa resulted in coarse, loose gels with high water loss.	[63]
Sodium-reduced chicken sausages	High-pressure processing (200 MPa, 10 min)	High-pressure processing contributed to increased firmness and decreased cook loss of chicken sausages. Colour parameters were lower in pressure-treated sausages. High-pressure processing treatment in combination with K-lactate or K-citrate reduced the perceived saltiness of the sausages.	[64]
Low-salt emulsified beef sausages	High-pressure processing (100–400 MPa at 10 °C for 15 min)	Lower salt (1.4%) in combination with high-pressure processing treatment, produced TVCs equivalent to the sample with 2.8% salt. Treatment at 200 MPa produces physicochemical properties similar to the sample with 2.8% salt. Sensory and textural properties of sausages decreased for high-pressure processing treatments ≥ 300 MPa.	[65]

## Data Availability

No new data were created or analyzed in this study. Data sharing is not applicable to this article.
